# Intracardiac echocardiography guided simultaneous atrial fibrillation ablation and Micra implantation 14 days after Watchman FLX implantation

**DOI:** 10.1002/ccr3.8906

**Published:** 2024-05-14

**Authors:** Ryuki Chatani, Hiroshi Tasaka, Shunsuke Kubo, Kenta Yoshida, Mitsuru Yoshino, Takeshi Maruo, Kazushige Kadota

**Affiliations:** ^1^ Department of Cardiovascular Medicine Kurashiki Central Hospital kurashiki Japan

**Keywords:** atrial fibrillation, catheter ablation, intracardiac echocardiography, leadless pacemaker, left atrial appendage closure

## Abstract

**Key Clinical Message:**

Atrial fibrillation ablation, including pulmonary vein isolation immediately after left atrial appendage closure (LAAC), is a rare and challenging issue. Intracardiac echocardiography guidance can help identify the LAAC device position for safe atrial fibrillation ablation without LAAC device‐related adverse events even immediately after LAAC device implantation.

**Abstract:**

Early phase atrial fibrillation (AF) ablation after left atrial appendage closure (LAAC) is a rare and challenging issue. Here, we present a case illustrating the feasibility of AF ablation under intracardiac echocardiography guidance immediately after LAAC device implantation without LAAC device‐related adverse events.

## Graphical abstract

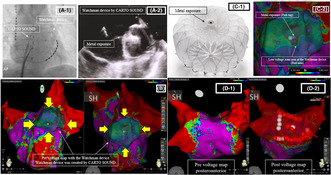

## 1 INTRODUCTION

Percutaneous left atrial appendage closure (LAAC) may be performed for patients with atrial fibrillation (AF) who are not suitable for long‐term anticoagulant therapy. Catheter ablation therapy is effective for drug‐resistant AF. Although the feasibility and safety of left atrial catheter ablation procedures in the presence of LAAC device implantation for the late phase >190 days have been reported,^1^ outcomes for the early phase <190 days remain unclear. Here, we report safe performance of intracardiac echocardiography (ICE) guided left atria catheter ablation in the early phase after left atrial appendage device implantation (after only 14 days) and Micra implantation.

## 2 CASE REPORT

### 2.1 Case history

A 70 year‐old woman with a history of ischemic stroke due to paroxysmal AF (PAF) and high bleeding risk (CHADS₂ score 3, CHA₂DS₂–VASc score 5, HAS–BLED score 4) underwent LAAC with a 31 mm Watchman flx (Boston Scientific). The left atrial appendage was successfully sealed and the patient was discharged on postoperative Day 2. However, on postoperative Day 6, she was rehospitalized due to bradycardia–tachycardia syndrome and PAF with syncope. When PAF stopped, a long sinus arrest of about 5 s was observed. A permanent transvenous pacemaker was considered; however, due to severe tricuspid regurgitation, a Micra device (Medtronic) was selected as a backup. The patient had undergone pulmonary vein isolation by cryoablation 1.5 years earlier and had not experienced recurrent PAF. However, due to strong palpitations with PAF, she requested simultaneous treatment with catheter ablation for second session. A second catheter ablation and Micra implantation were thus performed on postoperative Day 14.

### 2.2 Investigations and treatment

**FIGURE 1:**
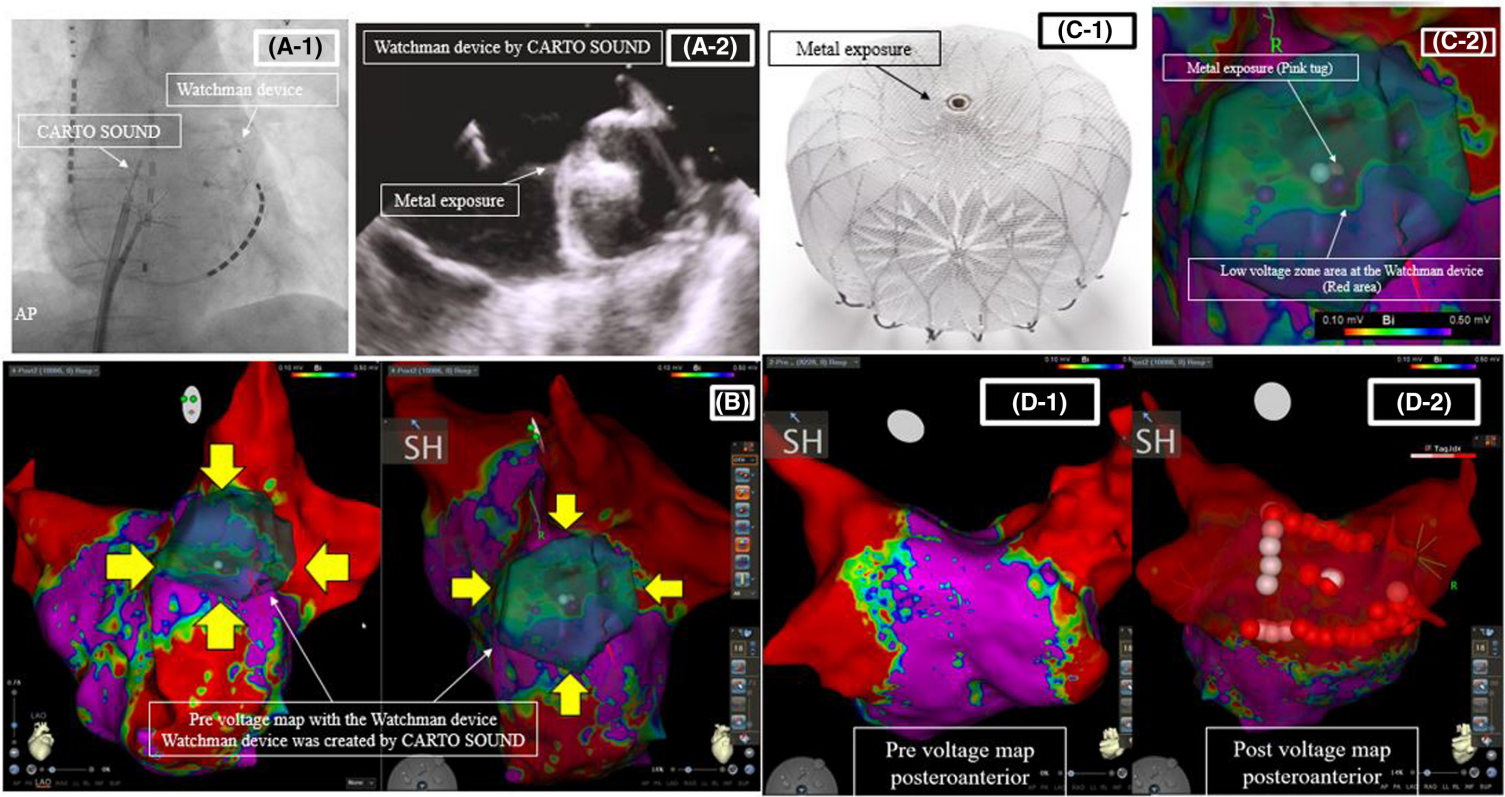
(A‐1) Fluoroscopic image of the CARTOSOUND and Watchman device. (A‐2) ICE image showing the Watchman from the left atrium. (B) Precatheter ablation voltage map with the Watchman (yellow arrows) created by CARTOSOUND. (C‐1) Watchman flx and metal exposure. (C‐2) The precatheter ablation voltage map around the Watchman device. (D‐1) The precatheter ablation map. (D‐2) The postcatheter ablation map.

**FIGURE 2:**
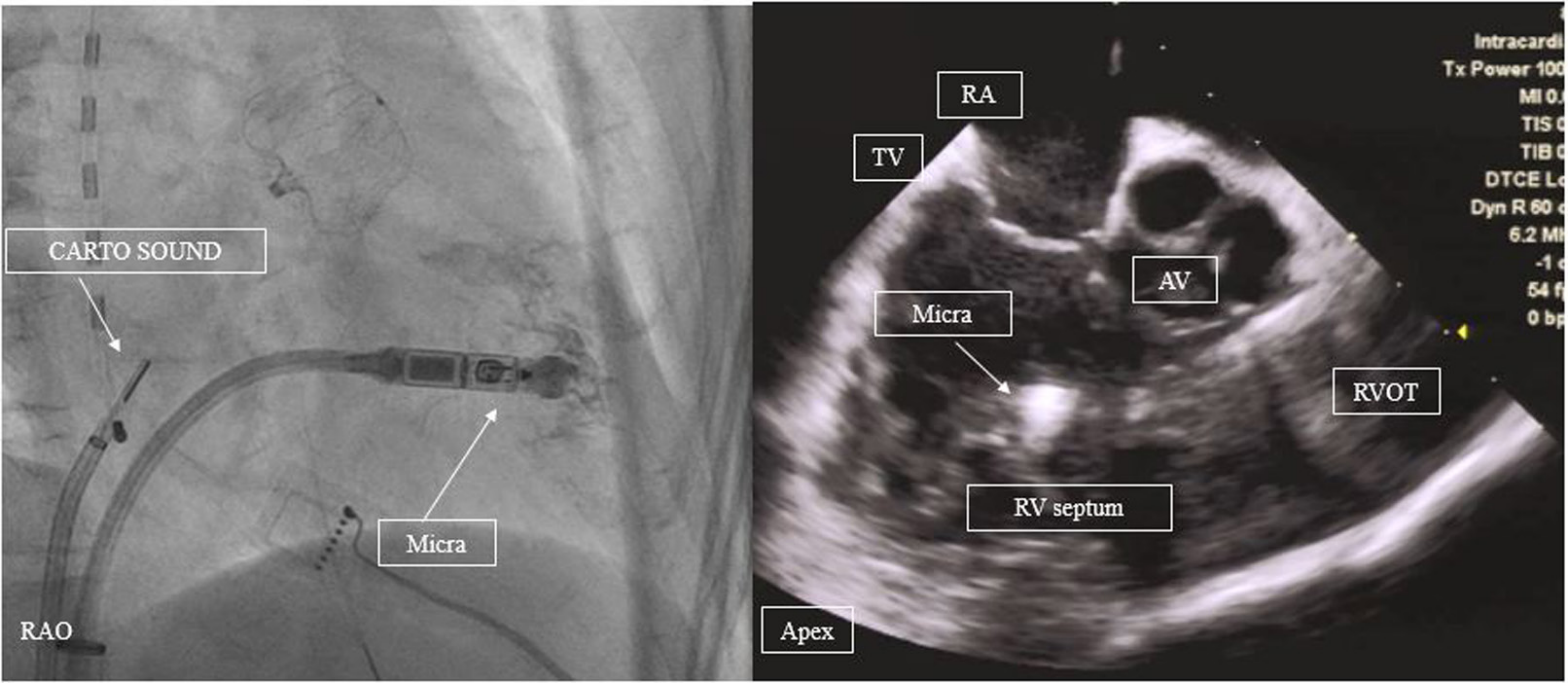
Fluoroscopic image of Micra from the right anterior oblique (RAO) view (left panel). ICE image showing Micra from the right atrium (right panel). TV, tricuspid valve; AV, aortic valve; RA, right atrium; RV, right ventricular; RVOT, right ventricular outflow tract.

Preoperative transesophageal echocardiography or cardiac computed tomography imaging was omitted due to urgency, and the procedure was started with the intention of stopping in case of major peri‐device leakage (>5 mm) or device‐related thrombosis on ICE imaging. First, CARTOSOUND (Biosense Webster) was used to confirm no peri‐device leakage or thrombi on the surface around the Watchman (Video S1). Watchman's map was created using CARTOSOUND (Figure 1A,B, Video S2). Despite reports of a normal bipolar voltage zone area (≥0.5 mV) following subendothelium of the Watchman,^2^ this case presented a reduced voltage zone area (<0.5 mV) around the metal exposed area, likely because it was immediately after device implantation (Figure 1C). Pulmonary vein reconnection was observed in the left pulmonary vein carina and posterior to the right lower pulmonary vein and additional ablation was performed. Given that this was a recurrent case with strong palpitations, posterior wall isolation was added to increase the long‐term non‐recurrence rate^3^ (Figure 1D). It should be noted that the patient had renal dysfunction (eGFR: 42), and there is a prior report of the effectiveness of ICE reducing the contrast media volume.^4^ Subsequently, Micra was successfully placed under ICE guidance to ensure that Micra did not face the free wall of the right ventricle, with a small amount of contrast medium (7 mL) (Figure 2). After the sheath was removed, the procedure was completed with a figure‐of‐eight suture. Micra™ pacing rate was set to 40 bpm with VVI mode. The procedure time was 124 min, and the patient was uneventfully discharged on the fifth day after the procedure.

### 2.3 Outcome and follow‐up

Her palpitations disappeared 3 months after discharge and warfarin was changed to aspirin 100 mg due to unknown anemia (hemoglobin: 9.2 g/dL). One month after the drug change, transesophageal echocardiography revealed no peri‐device leakage or device‐related thrombosis and anemia improved (hemoglobin: 13.3 g/dL).

## 3 DISCUSSION

Robust endothelialization of the Watchman device atrial surface by 28 days has also been reported in a canine model,^5^ suggesting tissue ingrowth as a source of chronic stability, but this is known to be delayed in humans. In this case, the degree of subendothelialization was evaluated by bipolar voltage in the early phase (after 14 days), suggesting that the subendothelialization of the central metal exposure was delayed. However, regarding endothelialization, in addition to changes over time, there may also be influences, such as the device type and position, pressure contact with the LAA wall, and rhythm, but this requires further investigation, including assessment of the above factors. Even when LAAC and left atrial catheter ablation are performed simultaneously, they should be preceded by catheter ablation to prevent device‐related complications, including device dislodgment, device‐related thrombosis, and exacerbation of peri‐device leakage. A previous study reported that adding LAAC to AF ablation did not increase the recurrence rate.^6^ However, even if AF ablation is performed before LAAC, a second AF ablation may be necessary if AF recurs. To our knowledge, there are no reports of left atrial catheter ablation in the early phase after LAAC device implantation. An increase in peri‐device leakage has been reported with left atrial catheter ablation treatment after LAAC,^7^ device‐related thrombosis has been reported to occur in 12.5% of patients after left atrial catheter ablation.^8^

Theoretically, performing extensive radiofrequency ablation and catheter manipulation in the left atrium in the presence of the LAAC device may increase the risk of various complications, including device dislodgment, device‐related thrombosis, and exacerbation of peri‐device leakage. Furthermore, catheter‐device contact and delivery of radiofrequency energy on the LAAC device surface may result in device dislodgment, subsequent thrombus formation, and peri‐device leakage. A previous study reported that patients who underwent LAA isolation had a much higher risk of new peri‐device leakage, and some patients had larger (>5 mm) peri‐device leakage.^7^ In addition, a previous study reported that the application of radiofrequency near the LAAC device during ablation could result in mechanically induced endothelial injury and device endothelialization delay.^8^ This may increase the risk of thrombus formation at the site of endothelial injury. For AF ablation after LAAC device implantation, energy sources other than radiofrequency, and noncomplex ablations other than LAA isolation may be better to avoid device‐related complications.

However, in this case, we performed radiofrequency AF ablation because of the second session. When we perform radiofrequency AF ablation in the presence of the LAAC device, we believe it is important to pre‐assess the position of the LAAC device using the 3D mapping system and ICE before the ablation procedure. Knowing the location of the LAAC device in advance to avoid unnecessary contact and heat conduction between the LAAC device and ablation catheter may reduce these complications. This case was performed with the Watchman FLX device, and if an Amplatzer Amulet device is used, the device disk may overlap the left pulmonary vein ridge and interfere with ablation of the ridge, including pulmonary vein isolation.

## 4 CONCLUSION

This case supports that catheter ablation for patients with symptomatic AF in the early phase after Watchman device implantation is feasible and safe under ICE guidance.

## AUTHOR CONTRIBUTIONS

**Ryuki Chatani:** Conceptualization; data curation; formal analysis; investigation; methodology; project administration; resources; software; supervision; validation; visualization; writing – original draft; writing – review and editing. **Hiroshi Tasaka:** Methodology; validation. **Shunsuke Kubo:** Conceptualization; methodology; validation. **Kenta Yoshida:** Methodology; validation. **Mitsuru Yoshino:** Methodology; validation. **Takeshi Maruo:** Methodology; validation. **Kazushige Kadota:** Methodology; validation.

## FUNDING INFORMATION

None.

## CONFLICT OF INTEREST STATEMENT

The authors have no potential conflict of interest relevant to this article.

## CONSENT

Written informed consent was obtained from the patient for the publication of this case report.

## Supporting information


Video S1.

Video S2.

## Data Availability

The data that support the findings of this study are available from the corresponding author upon reasonable request.
